# Eliminating invisible deaths: the woeful state of global rabies data and its impact on progress towards 2030 sustainable development goals for neglected tropical diseases

**DOI:** 10.3389/fitd.2024.1303359

**Published:** 2024-03

**Authors:** Catherine Swedberg, Katrin Bote, Luke Gamble, Natael Fénelon, Alasdair King, Ryan M. Wallace

**Affiliations:** 1 Poxvirus and Rabies Branch, Division of High Consequence Pathogens and Pathology, US Centers for Disease Control and Prevention, Atlanta, GA, United States; 2 Department for Neglected Tropical Diseases, World Health Organization, Geneva, Switzerland; 3 Mission Rabies, Cranborne, United Kingdom; 4 Communicable Diseases and Environmental Determinants of Health, Pan American Health Organization, Port au Prince, Haiti; 5 International Veterinary Health, Merck Animal Health, Madison, NJ, United States

**Keywords:** data quality, dog rabies, disease burden, Integrated Bite Case Management, surveillance, Zero by 30

## Abstract

Like other neglected diseases, surveillance data for rabies is insufficient and incompatible with the need to accurately describe the burden of disease. Multiple modeling studies central to estimating global human rabies deaths have been conducted in the last two decades, with results ranging from 14,000 to 74,000 deaths annually. Yet, uncertainty in model parameters, inconsistency in modeling approaches, and discrepancies in data quality per country included in global burden studies have led to recent skepticism about the magnitude of rabies mortality. Lack of data not only limits the efficiency and monitoring of rabies elimination strategies but also severely diminishes abilities to advocate for support from international funding agencies. Meanwhile, the most vulnerable communities continue to suffer from deaths that could have been prevented through more robust reporting. The Zero by 30 global strategy to eliminate dog-mediated human rabies by 2030 recommends endemic countries adopt the intersectoral approach, Integrated Bite Case Management (IBCM), as a cost-effective method to enhance surveillance. However, effective implementation of IBCM is impeded by challenges such as limited capacity, resources, knowledge, skills, and attitudes toward compliance. To address this, the World Health Organization and United Against Rabies Forum have developed several open-access tools to guide national control programs in strong data collection practices, and online data repositories to pragmatically streamline reporting and encourage data sharing. Here, we discuss how current and future initiatives can be best employed to improve the implementation of existing surveillance tools and prioritization of effective data reporting/sharing to optimize progress toward 2030 elimination.

## Introduction

Rabies is one of 21 conditions listed by the World Health Organization (WHO) as a neglected tropical disease (NTD) (1). NTDs encompass a wide variety of causative agents (e.g., bacteria, parasites, viruses), geographic ranges (e.g., foci of Guinea Worm infections to near global endemicity of rabies), and health outcomes (e.g., long-term disabilities to invariable death). On paper, many of these NTDs may appear to have nothing in common; many would struggle to identify commonalities between Hansen’s disease (bacteria), Chagas (protozoan), snake envenoming (toxin), Guinea Worm (parasitic) and Rabies (viral zoonoses). However, all NTDs share one primary underlying attribute that these diseases are often associated with poverty, limited access to healthcare, and inadequate sanitation and housing (1, 2). While the pathogenesis of and interventions for the control of NTDs may have few similarities, NTDs all suffer from the same root causes of their persistence; neglect for the control of diseases and the health of the populations they affect.

While the term “NTD” implies that there is little effort or interest in controlling these diseases, WHO and partners have successfully established several initiatives focused on reducing the burden of NTDs. The first NTD Roadmap was launched on January 30th, 2012 (3), with that day officially recognized in 2020 as World NTD Day by WHO’s World Health Assembly to increase awareness of the devastating effects and support the momentum. On the same day in 2021, the new NTD Roadmap for 2021 to 2030 was published, setting global targets aligned with the Sustainable Development Goals (SDGs) (4). Prevention, control, elimination, or eradication of NTDs are intertwined with many of the SDGs in addition to the inclusion of the SDG Target 3.3 to “end the epidemics of neglected tropical diseases” by 2030, as part of Goal 3 (Ensure healthy lives and ensure well-being for all at all ages) (5). For example, SDG 1 (No poverty), SDG 4 (Quality education), SDG 6 (Clean water and sanitation), SDG 10 (Reduced inequalities), and SDG 11 (Sustainable cities and communities), all influence NTD prevention and control programs, such as those for rabies (6, 7).

In 2015, Rabies was the first zoonotic NTD to be designated for “Elimination as a Public Health Problem” as a part of the Tripartite’s commitment (8). This declaration resulted in the development of the *Zero by 30* (ZB30) global strategy to end human deaths from dog-mediated rabies by 2030 (9). Since essentially all high-income countries have already eliminated dog rabies, ZB30 objectives specifically target 100 low- and middle-income countries (LMICs) that still endure human rabies deaths transmitted by dogs. Impetus from ZB30 has built a strong foundation for rabies elimination by catalyzing the development of numerous tools and initiatives to increase the prospects of reaching goals in endemic LMICs. In 2020, the United Against Rabies (UAR) Forum was launched as an inclusive network aiming to bring together stakeholders from different sectors, following a One Health approach to foster collaboration and offer support to accelerate achieving ZB30 goals (10, 11).

Unfortunately, early successes in the global initiative to amplify capacity for rabies control programs were thwarted by the COVID-19 pandemic, which saw many rabies vaccination and surveillance programs halted (12–14). As these critical rabies programs regain momentum post-COVID emergency, there is an opportunity to re-examine the primary barriers to achieving ZB30 and refocus efforts to maximize the likelihood of eliminating of one of the oldest diseases known to man. Here, we discuss how current and future initiatives can be best employed to improve the implementation of existing surveillance tools and prioritization of effective data reporting/sharing to optimize progress toward 2030 elimination goals.

### The root cause of rabies neglect

It is difficult to point to one universal cause of the persistence of dog-mediated human rabies deaths in over 100 countries; the disease is zoonotic, control efforts must consider stray dog vaccination programs, dogs have little economic value unlike livestock, the disease impacts people in marginalized communities who cannot access healthcare services, and this list could go on. In this article, we have chosen to focus on what many learned in Epidemiology introduction courses regarding data and policy—“Garbage in, Garbage out.” This phrase emphasizes how the lack of accurate and quality data limits the development and implementation of effective policies and feeds the Cycle of Neglect. To directly tie this core epidemiologic principle to rabies: (1) if communities are not capable of detecting human rabies cases, then (2) deaths will go undocumented by health systems and stakeholders/nations cannot prove the burden of disease. Therefore, (3) policymakers have little incentive to invest public resources into the control of this disease.

### What happens when someone dies of rabies?

To truly understand how the Cycle of Neglect is pervasive for rabies, we should explore the unique pathophysiology of this virus that not only helps it hide within the human body, but also within communities, countries, and the world. Rabies is one of the few neurotropic viruses that impacts mammals (15). Neurotropic viruses enter then target the nervous system, where mammals are unable to mount an immune response. In effect, the virus conceals itself from the immune system as it is transported from the location of exposure to the brain.

This unique process of infection leads to several key challenges when trying to identify rabies victims. First, persons are completely asymptomatic during the greater part of their infection, with clinical signs only appearing after the virus has begun replicating within the neurons of the Central Nervous System (16, 17). Second, there are no tests to determine if someone has rabies virus infection until they have begun showing signs, with post-mortem testing of the brain often required. Moreover, tests currently used for human rabies require advanced pathology and laboratory services, and most post-mortem testing typically requires training and skills for invasive sample collection. Lastly, the onset of clinical signs is sudden, non-specific, and invariably fatal. This means that rabies patients are frequently misdiagnosed as other more common acute febrile diseases such as cerebral malaria or viral meningitis (18). Rabies virus is a master of invisibility, both within the body of its victims and within the communities it impacts.

### Who is dying from rabies?

Referring back to the definition that unites the NTD community—Rabies victims are often members of marginalized, poverty-stricken communities that not only lack access to affordable fundamental healthcare but also lack advocacy for the control of the many afflictions they face (6). Unlike the political decisions for allocating healthcare resources, the rabies virus does not discriminate. The virus will readily infect any mammal, including humans, and does not contemplate socioeconomic factors such as level of education, wealth, prestige, power, or status attained. So, if the virus is not genetically adapted to infect the marginalized, then why are the deaths so concentrated in these communities? The answer lies in the level of access to affordable life-saving human rabies vaccines that must be administered shortly after a rabies exposure occurs to prevent infection (19). Furthermore, the populations with the highest burden typically do not have sustained funding for rabies awareness campaigns or annual dog vaccinations to reduce rabies in the reservoir dog population.

Most rabies-endemic LMICs have established government-operated clinics dedicated to providing human rabies post-exposure prophylaxis (PEP). However, these clinics are often centralized, located in urbanized areas and at primary health centers, which marginalized and impoverished communities cannot easily access (20–22). In an ideal situation, rabies biologicals like vaccine and immunoglobulin (i.e., PEP) would be adequately stocked in smaller secondary and tertiary clinics in rural communities at an affordable cost to ensure wider availability and accessibility to disadvantaged populations. In many settings, when government-procured low-cost vaccines are not routinely available, the only option for those bitten or scratched by dogs is to seek high-cost vaccines from private pharmacies (23, 24). Persons with knowledge of post-exposure healthcare recommendations and the ability to readily access these clinics will almost certainly seek and receive PEP to survive rabies exposure. Not surprisingly, the community members with these advantages tend to be those who are highly educated and wealthy.

Moreover, we must not forget that there is always a cost associated with receiving medical care. The cost to patients for rabies vaccines is highly variable and directly related to the method of administration used (intradermal vs intramuscular), the regimen recommended, and the reimbursement policy of the country (government-provided vs patient-purchased) (24, 25). In settings where patients must pay for vaccines on their own, through the intramuscular route, costs can be as high as USD 100 per course (21, 26), which is a typical month’s salary for many low-income families. But even if the vaccine is administered in a low-cost manner (government-supported and intradermal), rabies vaccination consists of three to five healthcare visits. This means low-income families have the additional burden of having to take time from work, travel potentially long distances (multiple times), and pay for wound care associated with the exposure. Healthcare access is a human right (27, 28), but until this right is realized for all communities, the rabies virus will continue to impact persons with unequal access to resources.

### Who is left to advocate for rabies?

Rabies leads to the inevitable death of anyone who does not receive PEP in time. There are only a handful of rare (and often disputed) reports of humans surviving rabies following the manifestation of disease (29, 30). Likely, there are no rabies survivors residing in communities to remind policymakers of the pain and suffering they endured during their illness. Rabies victims have sudden onset of symptoms, rapid progression to coma, and death within days to weeks (16, 17). While many NTD victims may spend years post-illness advocating for disease control, a rabies victim has mere days. It is often proposed that families can be advocates for rabies victims. However, with most rabies cases being children under the age of 15 (6) and the horrendous way rabies victims often die, the trauma of these events can be all too unbearable for a family. Given this, it is easy to understand why a family may not wish to relive this trauma in the hopes of advocating for rabies control.

Many NTDs cause long-term disabilities and unaccounted psychological suffering (31). Lymphatic filariasis causes severe and painful swelling of limbs that rarely go unnoticed by members of the community in which they reside. Guinea Worm causes an incredibly painful eschar that can lead to people spending days or weeks out of work. Hansen’s disease causes the slow and painful necrosis of connective tissue, commonly leaving people with disfigured faces, not to mention social stigma and exclusion. Most NTDs do not lead to the victim dying but rather relegate these victims back into their communities, as visual reminders that the disease is present and could affect anyone unfortunate enough to become exposed (32). With diseases such as these, past victims become advocates for their control, whether they desire that role or not. For rabies, while inevitable death prevents victims from being advocates themselves, those who survive rabid dog exposures are one of the few voices able to illuminate the often-unseen suffering caused by this virus to policymakers.

### Conflicting estimates for the burden of rabies

If one could design the ideal pathogen to evade elimination efforts, it may look very similar to rabies virus. As previously stated, this pathogen hides from the body’s defense system, hides from the community, and does not leave any victims alive to serve as a warning to others. From a public health perspective, detection and documentation of cases are critical to understanding the disease burden, which is a key factor in prioritizing support for healthcare interventions. Given the woes of human rabies case detection, advocacy organizations in the rabies community have had to resort to relying on epidemiological models to estimate the disease burden. This presents several key issues when trying to advocate for rabies control. First, it may be difficult for politicians to support disease prevention that only exists as a hypothetical estimate of human mortality output from published models. Second, the results from published models describe a conflicting landscape of uncertainty and range of estimated burden caused by rabies.

The first attempt to quantify the burden of rabies globally was conducted by Knobel et al. in 2005 who reported that an estimated 55,000 people die from rabies annually (33). This estimate was updated in 2015 with a new study that found a relatively congruous value of 59,000 human rabies deaths annually (upper confidence limit of 175,000 deaths)! (34). In 2019, a similar modeling study was published that increased this estimate to ~74,000 deaths (35), in accord with the upper 95% confidence interval estimated by Bonaparte et al. in 2023 (36). While most global burden studies have been generally consistent, we must acknowledge that the uncertainty in these models is vast with death estimates ranging from 20,000 to 175,000 per year. Recently, a different study methodology was used to estimate the global burden which concluded only 14,000 human deaths occur each year (37). Modeling disease burden is an unfortunate necessity when the ability to detect true cases is lacking. Yet, modeled rabies deaths have not been effective at motivating policymakers, with inconsistent and questionable burden estimates only making the task of advocating for this NTD that much more challenging. Whether the true number of human deaths is 14,000 or 55,000 or 74,000, the fact remains that fewer than 1,500 of these deaths are reported to health officials each year (38).

### Improving rabies surveillance

Implementing effective rabies surveillance has the duality of being as straightforward as it is complicated. Due to a low incidence and short time window for diagnosis, detecting rabies in animals can be like looking for a needle in a haystack. Rabies surveillance systems ideally comprise three functionalities: (1) Rabies Exposures (2) Animal Rabies and (3) Human Rabies (39). In recent years, international agencies have released updated guidance on ideal surveillance systems, which link Exposures and Animal Rabies (6, 9) to prevent people from developing rabies and entering the “Human Rabies” system. Termed Integrated Bite Case Management (IBCM), this intersectoral approach to finding people with rabies exposures before the onset of symptoms, and thus death, has become a key strategy for achieving ZB30 (40–43).

IBCM operates by using health facilities as sentinels to assess for the risk of rabies in presenting bite patients to identify potential exposures, then investigating the animal(s) involved. During this investigation, healthy animals are observed for signs of rabies for 10 to 14 days, with any dead or euthanized animals tested immediately. Through this process, it can be determined if the patient experienced a true rabies exposure or a bite/scratch from a healthy animal, posing no risk of rabies. Countries can thereby use pre-existing systems to identify bite victims that require PEP to improve patient care and compliance, triage limited PEP supply for true exposures, and reduce human deaths even in the most marginalized communities. Over the last decade, implementation of IBCM in low-resource settings, like Haiti, has shown to improve health outcomes and be more cost-effective than rabies control programs not using an IBCM approach (44).

Innovation and funding have enabled many endemic LMICs to implement IBCM programs (45) and deploy new point-of-care diagnostic tests for suspected rabid animals. These advancements are expected to further enhance animal rabies surveillance systems in the coming years. To date, there are at least six commercially available point-of-care tests for animal rabies diagnosis, but only two have demonstrated high sensitivity, and none have undergone formal evaluation for recognition by WHO or World Organisation for Animal Health (WOAH) (46). Therefore, point-of-care test results should not influence PEP decisions and laboratory confirmation with WHO/WOAH-recommended testing (e.g., direct fluorescent antibody, polymerase chain reaction) is still required. Moreover, no such innovation, effort, or funding has been identified to improve human rabies surveillance systems. To strengthen detection of human and animal cases, the rabies community must unite to prioritize feasible ways to overcome these challenges.

### WHO initiatives for data reporting and sharing

The foundation of any surveillance system is a consistent and accurate case definition. For human rabies, WHO has the role of developing and disseminating case definitions that should be incorporated into every rabies program (6). However, a recent UAR Stakeholder Event held in Paris (2022) resulted in participants documenting numerous frustrations with conflicting rabies case definitions and an overwhelming number of rabies “guidance documents” from multiple organizations (47). To bring clarity to the rabies community, WHO and UAR released a comprehensive guidance document focused solely on disseminating the official case definitions and minimum data elements (48, 49) that are required to be submitted to WHO’s Global Health Observatory (38). Additionally, WHO has now produced and disseminated multiple advocacy materials to help countries implement these definitions and is in the process of revamping its online data repository for public open access to key rabies metrics (50–52). All of these tools are moot unless governments and health systems improve their ability to detect rabies deaths (53). Nevertheless, when these systems eventually improve, WHO platforms will be ready and able for receipt of data to assess the global rabies burden and monitor progress toward ZB30.

### Gavi support for human rabies vaccine

Gavi, the Vaccine Alliance, is a global partnership aiming to increase access to immunization in low-income countries by supporting the introduction and distribution of vaccines (54). In 2018, the Gavi Board approved a Vaccine Investment Strategy (VIS) for the period 2021 to 2025 (55), with the inclusion of human rabies vaccines for the first time—a significant development in the implementation of ZB30. While the emphasis of Gavi temporarily shifted toward establishing and supporting the COVID-19 Vaccines Global Access (COVAX) platform during the COVID-19 pandemic, the Gavi Board has now decided to resume the implementation of new vaccine programs outlined in their VIS (56). The planning for the rollout of rabies vaccines has already commenced, with eligible endemic countries expected to be able to apply for support as early as mid-2024.

## Conclusion

Numerous initiatives have been established to propel the world towards eliminating dog-mediated rabies. Notable among these are the development of the UAR Forum (10) and new public-private partnerships like the International Rabies Taskforce (IRT) (57), which in 2022 vaccinated over 500,000 dogs in LMICs. While these initiatives should serve as inspiration to the rabies community, they are still a proverbial ‘drop in the ocean’ from a global elimination perspective. At the current trajectory, one of two outcomes is likely to transpire over the next decade of rabies control. Optimistically, rabies vaccination programs will experience an exponential scale-up and we will see rabies eliminated before we ever actually know just how many people die from this disease. Alternatively, enthusiasm for funding and a workforce to operate large-scale dog vaccination programs will dwindle as policymakers and funders question the impact of such programs. To avoid a more somber outcome, stakeholders in the rabies community must invest in alternative means to diagnose patients with encephalitis, improve healthcare system access for marginalized communities, and encourage governments to establish functional reporting systems and share their data. As the 2030 goal is fast approaching and core functions of rabies elimination have been known for decades, the time for overly ambitious and comprehensive rabies planning has ended, and the era of practical implementation must begin now.

## Data Availability

The original contributions presented in the study are included in the article/supplementary material. Further inquiries can be directed to the corresponding author.
